# Percutaneous tracheostomy in COVID-19 patients: a retrospective cohort study

**DOI:** 10.1590/1516-3180.2024.0036.R2.07032025

**Published:** 2025-08-29

**Authors:** Sofia Wagemaker Viana, Brenda Feres, Gabriel Roberto, Rodrigo Sardenberg

**Affiliations:** I Physician. Kursk State Medical University (KSMU), Kursk, Russia.; II Physician. Kursk State Medical University (KSMU), Kursk, Russia.; III Physician. Center for Advanced Research at Union of Great Lakes University, São Paulo (SP), Brazil.; IV Professor; Physician. Hospital Alemão Oswaldo Cruz, São Paulo (SP), Brazil.

**Keywords:** Tracheostomy, COVID-19, SARS-CoV-2, Thoracic surgery, Percutaneous tracheostomy, Patient care, Brazil

## Abstract

**BACKGROUND:**

The coronavirus disease 2019 (COVID-19) pandemic has placed unprecedented strain on healthcare systems, particularly on critically ill patients requiring prolonged mechanical ventilation (MV). Percutaneous tracheostomy (PT) has emerged as a potential strategy to facilitate weaning, reduce intensive care unit (ICU) stay, and optimize resource use. However, the timing, safety, and outcomes of PT in COVID-19 patients remain debatable.

**OBJECTIVES:**

This study aimed to describe the technical aspects of the procedure and evaluate the early safety of our technique to healthcare professionals, as well as the short-term factors affecting survival in 103 consecutive patients after tracheostomy.

**METHODS:**

We retrospectively analyzed patients with COVID-19 who underwent PT between March 2020 and June 2020 at Hospital Alemão Oswaldo Cruz, São Paulo. The factors considered for analysis included age, sex, timing of tracheostomy, proportion of affected lungs, comorbidities, fraction of inspired oxygen on MV, and availability of professional private equipment. Univariate analysis was performed for screening, and variables with P < 0.20 were included in the multivariate Cox proportional hazards regression model.

**RESULTS:**

Most patients were male, with a median age of 68 years. The most common comorbidities were hypertension (n = 55/52%), diabetes (n = 37/36%), and heart disease (n = 24/21%). Patients over 60 years old had reduced survival (hazard ratio [HR] = 3.35; P = 0.003), and those who underwent high nasal flow catheter (HR = 0.49; P = 0.02) and PT earlier (< 10 days) had better survival (HR = 0.37; P = 0.04).

**CONCLUSION:**

Early PT in selected patients may reduce the duration of MV and lead to shorter ICU stays. The health system is overloaded by the scarcity of ventilators and beds for critically ill patients.

## INTRODUCTION

 Coronavirus disease 2019 (COVID-19) is a worldwide pandemic, with over 225 million cases diagnosed to date.^1^ While most COVID-19 patients do not require supportive care, 10–15% of patients develop acute respiratory distress that requires invasive ventilatory support.^2^ More than 190 countries registered cases that reached an outcome, and a total of 4.641.746 resulted in mortality.^1^ In Brazil, there were 31.895.385 COVID-19 cases and 561.762 deaths, with a 2.8% lethality rate. In São Paulo, the epicenter of the disease in Brazil, there were 4.113.741 confirmed cases and 140.677 deaths, with a lethality rate of 3.9%.^3^

 Mechanical ventilation (MV) for patients infected with severe acute respiratory syndrome coronavirus 2 (SARS-CoV-2) is associated with prolonged airway intubation and a high worldwide mortality of at least 50–67%.^4^ Based on previous experience with severe acute respiratory syndrome (SARS) in 2003, aerosol-generating procedures such as tracheal intubation or tracheostomy were considered high-risk procedures for the transmission of SARS-CoV-2 to healthcare professionals (HCP).^4^

 Tracheostomy is a common procedure in critically ill patients who require an extended period of MV. Use of tracheostomy can facilitate weaning from MV and potentially increase the availability of intensive care unit (ICU) beds.^4^ When the COVID-19 pandemic spread to Italy and Spain, ICUs experienced a massive influx of patients who were critically ill, many becoming candidates for tracheostomy. However, tracheostomy is an aerosol-generating procedure; therefore, HCP are at risk of infection during insertion and subsequent care, even when appropriate personal protective equipment (PPE) is used.^4^

 The American Academy of Otolaryngology and the ENT academy in the United Kingdom have stated that providers should "avoid tracheotomy in COVID-19 positive or suspected patients" because of the risks to HCP. These guidelines, based on limited evidence, recommend that tracheostomies should not be performed before 2–3 weeks after intubation, preferably after negative COVID19 testing, and recommend open tracheostomy (OT) in these circumstances as opposed to percutaneous tracheostomy (PT).^4,5^

 The indications and ideal timing of tracheostomy in patients with COVID-19 remain controversial. Although several studies have been published with recommendations on proper and safe tracheostomy in these patients, the indications and timing of the procedure remain unclear and are mostly based on previous experience gained from the 2003 SARS-CoV-1 epidemic.^6,7^

**Figure 3:**
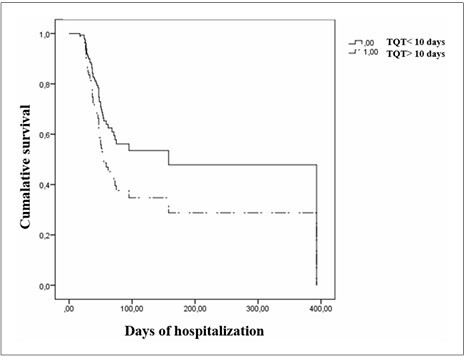
Cumulative survival in individuals who underwent tracheostomy before 10 days before orotracheal cannula (HR = 0.54; P = 0.2).

 A recently published report has discussed the timing of tracheostomy in COVID-19 patients. The authors emphasize that tracheostomy reduces ICU stay and, in the context of prolonged MV, should be suggested within 7–14 days in order to avoid potential tracheal damage.^7^ Performing an early tracheostomy in a critical patient (before 10 days of MV) seems to reduce the risk of mortality and increase the probability of discharge from the ICU.^8^ Although it is a probability, some studies show promising results.^ 3,9^ In addition, according to our own experience, tracheostomy reduces mortality and sometimes influences discharge time (**Figure 3**). 

 In this manuscript, we describe the technical aspects of the procedure, evaluate the early safety of our technique for HCP, and identify short-term factors affecting survival in 103 consecutive patients after tracheostomy. 

## METHODS

 We retrospectively analyzed 103 consecutive patients with COVID-19 who underwent PT between March 10, 2020 and June 30, 2020, at Hospital Alemão Oswaldo Cruz, São Paulo. All the patients agreed to participate in this study. 

 This study included all mechanically ventilated patients who were both COVID-19 positive and received a tracheostomy consultation. Each patient was evaluated individually by the multidisciplinary team, and the appropriateness of tracheostomy was assessed by considering patient prognosis and goals of care, potential benefit of the procedure, and tolerability of the procedure. The main criteria for patient selection for tracheostomy were possibility of long MV time, comorbidities, positive end-expiratory pressure (PEEP) < 12 mmHg, and fraction of inspired oxygen (FiO _2_) < 70%.^3^

 The patients had SARS-CoV-2 infection documented by a nasopharyngeal swab for reverse transcriptase polymerase chain reaction (PCR) assay and developed severe respiratory failure requiring MV. Data were collected following a medical record review of each patient’s chart. This study was approved by the institutional review board (4.849.515). 

 Patients with the following characteristics were considered eligible for the analysis: (1) SARS-CoV-2 confirmed by testing, (2) MV due to COVID-19-related respiratory failure , and (3) underwent PT. 

 Several COVID-19-related factors were considered, including age, sex, timing of tracheostomy, type of technique, proportion of lungs affected, comorbidities, duration of and FiO_2_fraction on MV, availability of PPE, and current patient status. All phases of routine tracheostomy care were considered in the review: the perioperative step was preferable in the ICU, appropriate PPE was used, and ventilation was maintained during tracheotomy until cuff inflation and circuit reconnection; the postoperative step was closed, inline suctioning, closed circuit with high-efficiency particulate arrestance (HEPA) filter if on mechanical ventilatory support, and heat and moisture exchange (HME) when off ventilatory support.^3^

 The concurrent goals of these modifications were to mitigate the risk to the HCP while preserving the risk-benefit profile for patients and the feasibility and safety of PT. In addition, we collected variables such as O_2_saturation when entering the ICU, FiO_2_ at admission to the ICU, partial pressure of oxygen (PaO_2_)/FiO_2_ relation, SAPS 3 score, use of noninvasive MV, use of high-flow nasal catheter (HFNC), and total time of orotracheal tube administration before PT. 

 All patients underwent chest CT prior to PT to evaluate the extent of lung lesions by SARS-CoV-2, which was divided into three degrees: 25%–50%, 50%–75%, and > 75%. MV settings recommended for the procedure were a PEEP ≤ 12 mmHg, FiO2 ≤ 70%, respiratory rate ≤ 25 breaths per minute, and partial pressure of carbon dioxide ≤ 60 mmHg. Patients with multi-organ failure (MOF) were excluded. 

 All personnel involved in this procedure used full PPE, according to the following institutional policies: hair cover, N95 mask, surgical mask, face shield, gown, and two layers of gloves upon entering the room. Maximal oxygenation was performed before the procedure (FiO_2_ = 100%). 

 PT was performed in the ICU according to the usual technique, with all patients under sedation and using muscle blockers to avoid coughing and aerosolizing the virus. With the ventilator in the standby mode, we deflated the endotracheal tube (ETT) cuff and retracted the ETT into the proximal trachea/subglottis. Before the airway opening, bronchoscopy was performed for secretion suction and tracheal puncture. The ETT cuff was reinflated, and the ventilator was restarted. The path was dilated with the "PORTEX KIT" dilator (PORTEX Medical). Before insertion of the tracheostomy cannula, the ventilator was again set to standby mode and the ETT cuff was deflated. A PT tube Portex #8.0 was inserted into the trachea for all for females and #9.0 for all males. The bronchoscope was advanced through the tracheostomy tube to confirm the position and correct size of the tracheostomy tube and remove any blood or secretions from the airway. The ventilator was then connected and restarted once the circuit was closed.^3^

 Our tracheostomy team consists of:

- two thoracic surgeons (one for bronchoscopy);
- one intensive care physician and one nurse;
- one physiotherapist.

The endpoints for this study were the safety and feasibility of our bedside PT in mechanically ventilated patients with COVID19 in the ICU to bring to them a better prognosis and life expectancy; early patient outcomes as decreased complications, mortality, ICU stay, MV time, and early HCPs; time of tracheostomy; and factors affecting survival. 

### Statistical analyses

 The SPSS 22.0^®^ package for Windows (IBM Corp. Released 2020) was used. Patient characteristics were analyzed using frequencies and percentages for qualitative variables and medians and interquartile ranges for quantitative variables. Univariate analysis was performed for screening, and variables with P < 0.20 were included in the multivariate Cox proportional hazards regression model. The Cox model yielded hazard ratios (HRs) with 95% confidence intervals (95% CI), establishing the likelihood of death according to the number of tracheostomy days. 

## RESULTS

 The patients included in this review (n = 103) underwent PT in the ICU between March 2020 and June 2021. All patients underwent surgery performed by the same team using the same surgical protocol and technique. 

 The patient characteristics, including demographics, lung injury, and respiratory support type and measures, are shown in **Table 1**. Most patients were male, and the most frequent comorbidity (**Table 2**) was hypertension (n = 55/52%). The most common comorbidities associated with death were hypertension (n = 27) and heart disease (n = 16) (**Table 2**). 

**Table 1:** Patient demographics

**Variables**		**Total group n = 103**	**Early PT n = 14**	**Late PT n = 89**
Sex (male); n (%)		76 (70)	8 (57)	68 (76)
Age (years); median [25%–75%]		68 [57–77]	71 [60–90]	69 [56–77]
CT scan (%) involvement; n (%)				
	*25%–50%*	54 (52)	9 (64)	45 (51)
	*50%–75%*	31 (30)	3 (22)	28 (31)
	*> 75%*	14 (14)	1 (7)	13 (15)
	*No signs of COVID-19*	4 (4)	1 (7)	3 (3)
Comorbidities n (%)				
	*Hypertension*>	55 (53)	6 (43)	49 (55)
	*Obesity*	7 (7)	2 (14)	5 (6)
	*Diabetes*	37 (36)	2 (14)	35 (39)
	*Dyslipidemia*	14 (13)	0 (0)	14 (15)
	*Smoking history*	7 (7)	0 (0)	7 (100)
	*Heart disease*	24 (23)	3 (21)	21 (24)
	*Lung disease*	9 (9)	0 (0)	9 (100)
	*Kidney disease*	3 (3)	0 (0)	3 (100)
	*Thyroid disorder*	17 (16)	1 (7)	16 (18)
	*Neoplasia*	13 (13)	0 (0)	13 (100)
SAPS 3; median [25%–75%]		48 [41–55]	53 [37–63]	48 [43–55]
SpO_2_ at admission in hospital; median [25%–75%]		93 [90–95]	93 [89–95]	94 [91–96]
FiO_2_ at admission in ICU; median [25%–75%]		141 [108–215]	187 [114–217]	141 [99–236]
PaO_2_/FiO_2_ at admission in ICU; mediam [25%–75%]		93 [90–95]	93 [89–95]	94 [91–96]
Use of non-invasive MV; n (%)		60 (58)	7 (50)	53 (59)
Use of HFNC n (%)		35 (34)	1 (7)	34 (38)
Orotracheal cannula (days); median [25%–75%]		16 [13–19]	7 [6–10]	16 [14–19]
ICU before PT (days); median [25%–75%]		17 [14–21]	9 [7–10]	18 [15–21]
Days in hospital before PT; median [25%–75%]		20 [16–24]	10 [7–13]	21 [18–25]
Total time in MV; median [25%–75%]		33 [24–44]	22 [13–44]	34 [25–44]
Total days in hospital; median [25%–75%]		48 [37–73]	48 [37–69]	45 [25–92]

n = number of individuals; % = percentage of individuals; CT = computed tomography; SAPS = Simplified Acute Physiology Score; SpO_2_ = oxygen saturation; Fi = inspiratory fraction; ICU = intensive care unit; Pa = partial pressure; MV = mechanical ventilation; PT = percutaneous tracheostomy; HFNC = high-flow nasal catheter. Data are expressed as median and percentile (25%–75%) or as the number and percentage of patients. No data were missing.

**Table 2:** Univariate and multivariate analysis for death in tracheostomized patients in ICU

	**Univariate**		**Multivariate**	
	**HR**	**P value**	**HR**	**P value**
Sex	1.36	0.35		
Age (> 60 years/old)	**3.38**	**0.005**	**1.02**	**0.01**
Hypertension	1.13	0.66		
Diabetes	1.31	0.33		
Obesity	1.67	0.47		
Heart disease	**1.5**	**0.17**		
Lung disease	1.67	0.23		
CT findings	0.93	0.78		
SAPS 3	**1.02**	**0.13**		
SpO_2_	1	0.98		
FiO_2_	**1**	**0.2**		
PaO_2_/FiO_2_	0.99	0.39		
Non-invasive MV	**0.67**	**0.16**		
HFNC	**0.59**	**0.1**	**0.49**	**0.03**
Days in ICU before PT	1	0.74		
Days in hospital before PT	1	0.93	**0.49**	**0.03**
PT < 10 days	**0.54**	**0.2**	**0.4**	**0.04**

HR = hazard ratio; CT = computed tomography; SAPS 3 = Simplified Acute Physiology Score 3; SpO_2_ = oxygen saturation; Fi = inspiratory fraction; Pa = partial pressure; MV = mechanical ventilation; ICU = intensive care unit; PT = percutaneous tracheostomy; HFNC = high-flow nasal catheter. Dependent variable = days of hospitalization until outcome (hospital discharge or death).

 When admitted to the ICU, 15 patients required an FiO_2_ fraction of 100% on MV. The median relation PaO_2_/FiO_2_ in all patients was 141 (percentile range, 84–800), the median number of days of MV before tracheostomy was 16 (percentile range, 13–19 days), and 62% of patients underwent non-invasive MV. The median hospital stay before PT was 20 days (percentile range, 16–24 days), and the median hospital stay was 48 days (percentile range, 37–73 days). 

 The median time on MV prior to PT was 16 days (percentile range, 13–19 days) after initial intubation. The median total time of MV was 33 days (percentile range, 24–44 days). To date, none of the team members has developed any symptoms and/or tested positive for COVID-19. 

### Univariate analysis

Table 2 shows the results of the univariate Cox proportional hazards regression analysis of the factors associated with mortality. Survival time was defined as the total days of hospitalization. 

**Figure 1:**
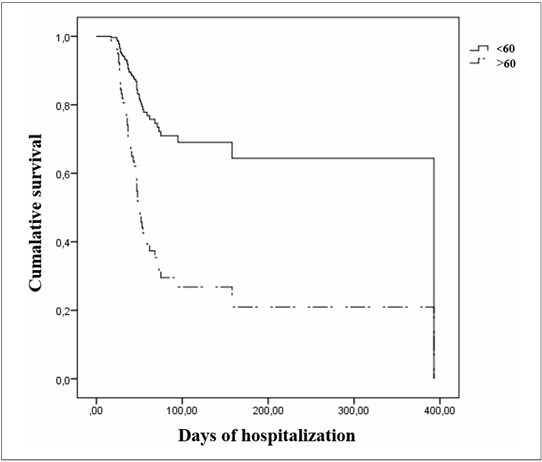
Cumulative survival according to age (HR = 3.38; P = 0.005).

**Figure 2:**
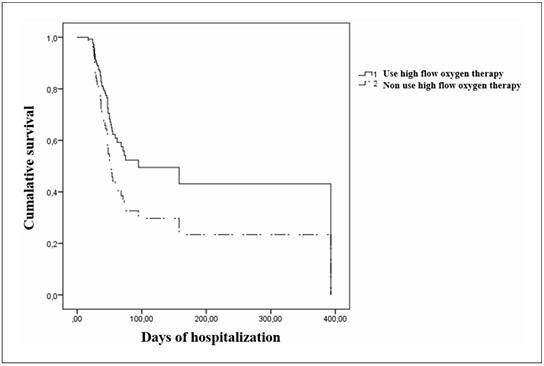
Cumulative survival according to the use of high flow nasal therapy (HR = 0.59; P = 0.1).

 Age, comorbidities such as heart disease, SAPS 3 prognostic score, use of HFNC, and number of days in the orotracheal cannula prior to tracheostomy were included in the multivariate analysis (P < 0.20). Older patients (> 60 years old) had a significantly decreased survival probability than younger individuals (HR = 3.38; P = 0.005) (**Figure 1**). In addition, it appears that patients who used oxygen in HFNC (HR = 0.59; P = 0.10; **Figure 2**) and those undergoing tracheostomy earlier (≤ 10 days) (HR = 0.54; P = 0.020; **Figure 3**) had a better survival probability than those who underwent tracheostomy later. However, these factors were not significant in univariate analysis (**Figure 2**,**Figure 3**). 

### Multivariate analysis

**Figure 4:**
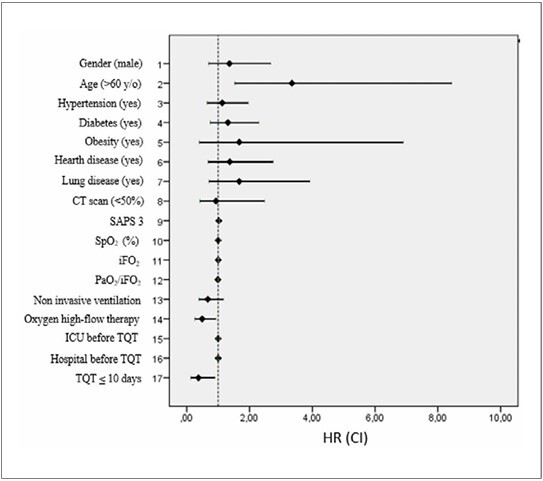
Hazard ratios and confidence intervals corresponding to multivariate analysis for age, use of oxygen high flow therapy, and days prior to tracheostomy; corresponding to univariate analysis for the other variables.

 In the multivariate analysis, patients over 60 years old had a reduced survival (HR = 3.35; P = 0.003; **Figure 4**). Patients who used HFNC prior to orotracheal intubation presented a better survival than those who did not (HR = 0.49; P = 0.02; **Table 2** and **Figure 4**). Patients who underwent PT earlier (< 10 days) also had better survival than those who underwent this procedure later (HR = 0.37; P = 0.04; **Figure 4**). 

## DISCUSSION

 The COVID-19 pandemic has pushed healthcare systems globally to their limits, with the unprecedented task of managing large volumes of critically ill patients. In this context, tracheostomy has emerged as an imperative component of care with a heightened risk of viral transmission to HCP and requires careful attention.^9,10,11,12^ Of primary concern in all countries is the relative scarcity of mechanical ventilators to support critically ill patients.^ 13,14^ This resource scarcity could lead to a push to perform tracheostomies. However, whether this would allow for a more expeditious ventilator weaning process remains unclear. 

 To the best of our knowledge, this is the largest sample from a single center published to date. The optimal timing of tracheostomy varies by clinical context. Outside of the current pandemic, it is generally recommended to be performed within two weeks post-intubation, as prolonged intubation is associated with post-intubation laryngotracheal stenosis.^6^ In our study, early tracheostomy patients had a better prognosis than late tracheostomy patients (**Table 1**). Currently, there is no evidence regarding the optimal timing of tracheostomy. 

 Two different tracheostomy techniques are currently available: OT and PT. The first is frequently performed in the operating room, although it can be performed at the bedside. No data are available to establish the superiority of one approach over the other in terms of infectious transmission or safety.^12,15^

 The role of identifying PCR test status in COVID-19 patients ahead of tracheostomy is unclear.^16,17^ Delaying tracheostomy to achieve negative test results is likely to prolong endotracheal ventilation and thus defer the potential benefits of tracheostomy while increasing the risk of complications related to endotracheal intubation.^17^

 PT may be performed by an intensive care specialist in the ICU; however, the thoracic surgeon normally participates in the procedure. It consists of a percutaneous tracheal puncture guided by bronchoscopy and progressive dilatation before insertion of the tracheostomy cannula, as described previously. A few complications have been described, including unexpected decannulation, wound infection, and postoperative bleeding.^6,7,11^

 Major complications of tracheostomy are rare. The risks of mortality, trachea-innominate fistula, and tracheoesophageal fistula from this procedure are all less than 1%. Early bleeding complications at the stoma are common (approximately 5%).^18^

 Although it is not possible to define the optimal timing, we believe that tracheostomy in a stable or clinically improved COVID19 patient should not be proposed before the 14th day after orotracheal intubation.^19,20^

 Early tracheostomy accelerates weaning from the ventilator and may have a critical role in freeing up ventilators, ICU beds, and staff during surges.^21^ This consideration is important, because resource scarcity may limit access to life-saving interventions for other patients.^22^ As shown by other authors, we argue that tracheostomy before 14 days has a role in a select group of patients with COVID-19 respiratory failure requiring prolonged MV. Figure 3 shows the cumulative survival in individuals who underwent tracheostomy before 10 days before orotracheal cannula. 

 The most important consideration regarding tracheostomy is not timing but whether the procedure is indicated. Critically ill patients requiring invasive ventilation have up to 50% mortality.^23^ There are several factors favoring early tracheostomy, including the cumulative dose of sedation, pulmonary hygiene, physical rehabilitation, airway complications, ventilator-associated muscle atrophy, ventilator-associated pneumonia, tracheal injury, ICU capacity in surge, and physical rehabilitation. However, there are factors that favor delayed tracheostomy, such as MOF, need for prone positioning, inability to tolerate the procedure (MOF), and risks to HCP.^23^

 Numerous randomized trials have demonstrated the benefits of early tracheostomy in appropriately selected patients. Tracheostomy reduces the cumulative sedation dose and allows for earlier participation in physical therapy and rehabilitation; this improvement in early mobility lessens the risk of critical illness myopathy.^24^ Early tracheostomy is also associated with earlier walking, talking, and eating.^25^ Earlier extubation lowers the risk of airway complications arising from prolonged translaryngeal intubation, such as focal tracheomalacia and tracheal stenosis. 

 Published information from the 2003 SARS outbreak showed that OT was the preferred technique. Current guidelines for choosing OT or PT for COVID-19 patients differ between countries based on each one’s preferences.^13^

 The 103 patients included in this study underwent PT by the same team using the same surgical protocol and technique. All procedures were performed at the bedside and no HCP involved in this process were infected. We emphasize the importance of being used to the procedure, making it safer and faster and minimizing viral exposure. 

 In our experience, the mortality rate among ICU COVID-19 patients undergoing tracheostomy was 30%, similar to the previously reported overall mortality rate. Deceased patients had at least one comorbidity (73% with cardiovascular problems and 36% with diabetes mellitus) and presented with > 75% lung affection. They were ventilated under FiO_2_ of 100% whenever entering the ICU, and had a mean total MV for 40 days. 

 Based on our data and those of previous publications, we could infer that tracheostomy did not affect the natural history of these patients. However, patients who underwent tracheostomy earlier (< 10 days) showed better survival. These findings may be related to fewer MV-related complications and early weaning from the ventilators, allowing these patients to leave the ICU before those who underwent the procedure later. In addition, these patients were in a more stable condition that allowed them to tolerate the procedure. These data were analyzed using multivariate analysis (P < 0.04). 

 Another interesting observation is the trend toward a significant association between the duration of intubation and overall survival; specifically, in our cohort, intubation longer than 20 days (median, 33 days) resulted in an increased risk of death. This result could support the choice to postpone OT in COVID-19 patients, as previously suggested for SARS-CoV-1 patients.^13^ We noticed better survival in patients who underwent HFNC and delayed or avoided the need for MV, which had an impact on outcomes in both univariate (P = 0.10) and multivariate analyses (P = 0.02). 

 As shown by other authors, the survival rate of COVID-19 patients requiring MV is extremely poor (< 30%). Otherwise, this argues against early tracheostomy.^20,26^

 Although it is not possible to define an optimal timing, we believe that tracheostomy in a stable or clinically improved COVID-19 patient should not be performed before the 7th day after orotracheal intubation.^20,27,28^

 Currently, evidence supporting a specific technique for tracheostomy in terms of minimizing the risk of HCP is lacking.^5^ There is no evidence to confirm whether the viral load of a patient at a specific time correlates with transmission risk to HCP. However, it has been shown that viral load does not correlate well with the severity of symptoms; therefore, not all critically ill patients will have high viral loads.^16^ Still, there is a consensus that providing adequate PPE for HCP is mandatory to mitigate infection in aerosol-generating procedures. 

 Some guidelines strongly advise that patients should test negative for COVID-19 before proceeding with tracheostomy, although the negative test should not diminish the exposure risk.^6^ Even in a recently published multi-society consensus statement, the writing panel could not find any evidence for recommending a specific tracheostomy timing in patients with COVID-19-related respiratory failure.^29,30^

 Early post-tracheostomy care is fundamental to minimize the risk of aerosol generation in HCP and other patients. Early provinces include keeping the cuff inflated, in-line airway suction, and aversion of humidified oxygen whenever feasible.^15^ The changing of the tracheostomy tube and progress on decannulation protocols should be judged case by case, since such procedures are also aerosol generators.^31^

 In our view, an enhanced level of PPE represents the safest possible level of protection for HCP and should be compulsory when performing PT. In our institution, complete PPE is available, and those involved in this process should receive PPE usage training, as this can represent a fount of contamination if not used properly.^29,30^

 Currently, the infection rate associated with tracheostomy is unknown. There is no evidence to support the use of a specific technique to minimize the risk of HCP exposure to airborne droplets. Although solid data on SARS-CoV-2 HCP infectivity are scarce, infection and death during HCP have been described.^31^

 A major limitation of our study is that the patients were recruited and compared from a single center. 

## CONCLUSION

 Our study demonstrated the feasibility and importance of early PT in COVID-19 patients. All 103 patients successfully underwent PT, and no major complications were reported. Tracheostomy may reduce the duration of MV and lead to shorter ICU stays. The health system is overloaded by the scarcity of ventilators and beds for critically ill patients, and the timing of PT should be taken into account. In conclusion, early PT in selected patients may reduce the duration of MV and lead to shorter ICUs stays. 
